# Using Google Trends for Influenza Surveillance in South China

**DOI:** 10.1371/journal.pone.0055205

**Published:** 2013-01-25

**Authors:** Min Kang, Haojie Zhong, Jianfeng He, Shannon Rutherford, Fen Yang

**Affiliations:** 1 Center for Disease Control and Prevention of Guangdong Province, Guangzhou, People's Republic of China; 2 Centre for Environment and Population Health, School of Environment, Griffith University, Brisbane, Australia; Northeastern University, United States of America

## Abstract

**Background:**

Google Flu Trends was developed to estimate influenza activity in many countries; however there is currently no Google Flu Trends or other Internet search data used for influenza surveillance in China.

**Methods and Findings:**

Influenza surveillance data from 2008 through 2011 were obtained from provincial CDC influenza-like illness and virological surveillance systems of Guangdong, a province in south China. Internet search data were downloaded from the website of Google Trends. Pearson's correlation coefficients with 95% confidence intervals (95% CI) were calculated to compare surveillance data and internet search trends. The correlation between CDC ILI surveillance and CDC virus surveillance was 0.56 (95% CI: 0.43, 0.66). The strongest correlation was between the Google Trends term of *Fever* and ILI surveillance with a correlation coefficient of 0.73 (95% CI: 0.66, 0.79). When compared with influenza virological surveillance, the Google Trends term of *Influenza A* had the strongest correlation with a correlation coefficient of 0.64 (95% CI: 0.43, 0.79) in the 2009 H1N1 influenza pandemic period.

**Conclusions:**

This study shows that Google Trends in Chinese can be used as a complementary source of data for influenza surveillance in south China. More research in the future should develop new models using search trends in Chinese language to estimate local disease activity and detect early signals of outbreaks.

## Introduction

Disease surveillance plays a key role in controlling and responding to influenza epidemics and pandemics [1]. Conventional surveillance for influenza is routinely recommended to monitor influenza-like illness (ILI) and influenza virus infections. Such surveillance involves the collection and analysis of data from clinics and laboratories. This traditional mode of surveillance is dependent on case reporting and medical records to track disease activity and time delays in reporting and case confirmation can slow detection of outbreaks or increases in influenza in the community. Thus epidemiologists have been investigating alternative data sources and real-time tools for influenza surveillance.

One new developing data source is internet search queries [2]. Every day, large numbers of users around the world search information via Web search engines. The internet search engine Google provides an internet service, Google Trends (GT), (http://www.google.com/trends/) for all internet users to browse the volume of search queries. GT analyzes a fraction of Google web searches to compute how many searches have been done for the terms that users enter, relative to the total number of searches done on Google over time [3]. It is reasonable that some GT regarding specific health issues can demonstrate the dynamic situation of internet health-seeking behaviors [3].

Google has found that some search queries related to influenza are good indicators of influenza activity. Not every user who searches for influenza information is actually sick, but a search spike probably indicates excess patients with symptoms of ILI searching online for information about influenza diagnosis and treatment. Hence Google developed Google Flu Trends (GFT) (http://www.google.org/flutrends/) in 2008 [4], to estimate national and regional influenza incidence. Some research in the United States has reported that GFT is highly correlated with historical ILI conventional surveillance data [5]–[7] and that this new tool can detect regional outbreaks of influenza 7–10 days earlier than the existing US CDC surveillance system [6]. GFT has now been applied in many countries, both at a national and sub-regional level [8]–[11]. However, neither GFT nor other search-term based tools for disease surveillance are available in China.

Guangdong province located in southern China is a semi-tropical region in Southeast Asia with a population of 100 million. Seasonal influenza occurs in Guangdong province annually. The pandemic H1N1 influenza (pH1N1) also affected the province heavily in 2009 [12]. The Guangdong provincial Centre for Disease Control and Prevention (Guangdong CDC), which is a WHO Collaborating Center for Surveillance of Emerging Infectious Diseases, has been conducting epidemiological and virological surveillance for influenza since 1998. Improving real-time surveillance in the community and hence improving influenza control is an important goal of Guangdong CDC.

As the results from elsewhere in the world suggest Google Trends is a useful surveillance tool, it is important to study whether this internet based tool is feasible for influenza surveillance in China. This paper examines the temporal correlation between Google Trends related to influenza and conventional surveillance data in Guangdong province to determine if an increase of web search matches actual influenza activity in this province.

## Materials and Methods

To measure the influenza epidemic situation in Guangdong, both influenza-like illness (ILI) surveillance and influenza virus surveillance are used by Guangdong CDC. ILI is defined as a fever ≥38°C with a cough and/or a sore throat. ILI surveillance consists of 56 sentinel clinics across the province. These clinics are also members of the national influenza surveillance system. They report weekly percentages of outpatients who present with non-specific signs and symptoms that meet a case definition of ILI. The second type of surveillance data is weekly laboratory test positive rates for influenza virus calculated by dividing the count for positive samples by the total number of specimens tested for influenza virus. This network consists of 22 laboratories across the province. For this study, surveillance data was provided by the Guangdong CDC.

There is no GFT for China or Guangdong, but GT data are available both at a national and provincial level. For computing how many searches have been done for the terms inputted, Google Trends analyzes a portion of Google web searches and normalizes the data to compare trends of different search terms from the same region during the same period. The GT data are displayed on a scale of 0 to 100 [13]. Based on common knowledge about influenza and the definition of ILI, we picked the terms *Flu, Common cold, Fever, Cough, Sore throat, Influenza A* and *H1N1*. Every selected term consisted of one translated word and its synonyms in the Chinese language. According to terms of use from Google, Google Trends can be used for education and research [13]. By setting the location parameter to “Guangdong, China”, and the time parameter to “2004-present”, we downloaded GT of all these search terms in Simplified Chinese separately from Google Trends (http://www.google.com/trends/).

As Google Trends for selected queries before 2008 was not available or was incomplete, our study period is from 2008 to 2011. ILI surveillance and GT data were collected for this period, but as the Guangdong CDC initiated weekly influenza virus surveillance in 2009, this type of data was only available for analysis for the period from 2009 to 2011. The units of analysis used were weekly percentage of clinic outpatients with ILI, weekly percentage of laboratory tests positive for influenza virus and weekly Google Trends. The study weeks are shifted by one day against those used for GT as the reporting week in the Google Trends data starts on Sunday, while the CDC surveillance week starts on Monday.

This study was approved by the ethics committee of the Guangdong Provincial Center for Disease Control and Prevention. There is no required written consent from patients as the study is based on aggregated and de-identified data. No information about the identity of any patients or internet users was retained.

Scatter plots were constructed to compare Guangdong CDC collected ILI and virus surveillance data with Google Trends data. From these comparisons, Pearson's correlation coefficients (*ρ*) with 95% confidence intervals (95% CI) were calculated for all the data and each calendar year separately. In previous research from the USA, it was found that Google Flu Trends could lead surveillance by one to two weeks [6]. Assuming a maximum lag of 2 weeks, we undertook lag correlation analyses to determine whether GT had a stronger association with lagged Guangdong CDC surveillance data. A relationship is reported as significant at p<0.05. All analyses were conducted using R, an open-source programming language for statistical analysis.

## Results

### Temporal trends

**Figure 1:**
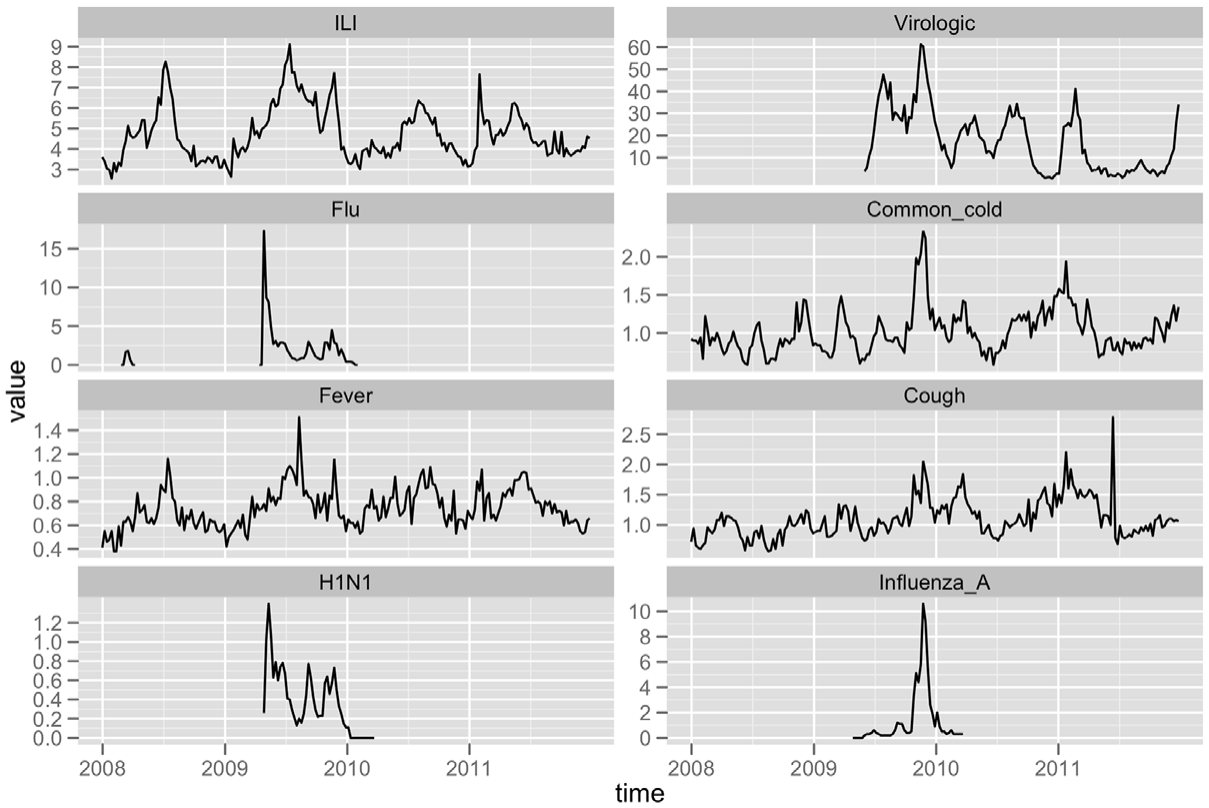
Time series plots of CDC Surveillance data and GT data in GD, from 2008 to 2011. The *ILI* and *virologic*alplots (top of the figure) are generated from CDC surveillance data for influenza. The *Flu, Common cold, Fever, Cough, H1N1* and *Influenza A* plots are based on data of Google Trends.

Our analyses used 209 weeks of data from 2008 to 2011, including 3 years of seasonal influenza epidemic data and 1 year of H1N1 influenza pandemic data. In 2008, 2010 and 2011, the ILI epidemic period was from week 5 (in February) through week 35 (in August), with a single peak in July or August. The highest weekly ILI percentage was 8.27%, 6.36% and 7.65% respectively in these three years of seasonal influenza. In 2009, the H1N1 influenza pandemic commenced in Guangdong province from May and there was an extra peak of ILI cases visits from Guangdong CDC data in November. The weekly ILI percentage ranged from less than 3% to more than 9%. The weekly positive rate of influenza virus was calculated from May 2009. The highest positive rate was 61.24% in November 2009, during the H1N1 pandemic period. In 2010 and 2011, the highest positive rate was 34.41% and 41.19% respectively (Figure 1).

From the time series of Google Trends data, there were more *Fever* searches during the warm season, from April to July, and more *Common cold* and *Cough* searches in the cold season, from December to February. Due to insufficient search volume, GT of *H1N1* and *Influenza A* were only observed in the influenza pandemic period. The *H1N1* search spiked in May 2009 while the peak for the *Influenza A* search was in November 2009 (Figure 1). Google Trends data for *Sore throat* was unavailable.

### Correlation analyses between CDC surveillance and GT

**Figure 2:**
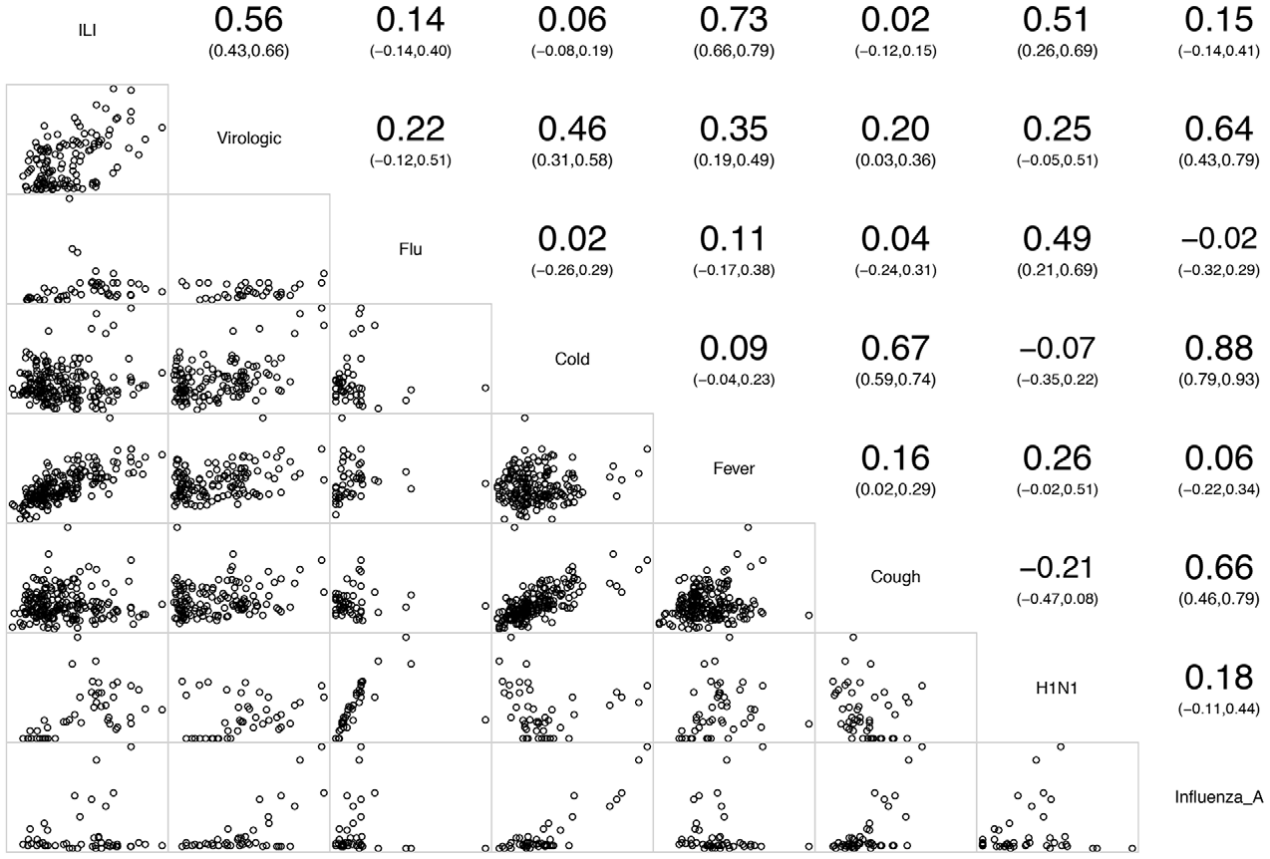
Scatter Plot and Pearson's Correlation Coefficient Matrix for comparisons among CDC Surveillance for influenza and Google Trends data, from 2008 to 2011. The upper panel above the diagonal shows Pearson's Correlation Coefficients with their confidence interval between CDC surveillance data and Google Trends data. The lower panel below the diagonal gives their scatter plots.

The virological surveillance data commonly had similar temporal patterns with the ILI in terms of peak incidence, with a correlation coefficient of 0.56 (95% CI: 0.43, 0.66) for the study period. Correlations between ILI and influenza virus surveillance and Google Trends varied based on the Google search terms used. The strongest correlation was between GT for *Fever* and ILI surveillance with a correlation coefficient of 0.73 (95% CI: 0.66, 0.79). The correlation between GT for *H1N1* and ILI surveillance was also significant, with a correlation coefficient of 0.51 (95% CI: 0.26, 0.69). When compared with influenza virological surveillance, the GT for *Influenza A* had a statistically significant correlation coefficient of 0.64 (95% CI: 0.43, 0.79). The GT for *Fever, Cold* and *Cough* also had statistically significant correlations with influenza virological surveillance data (Figure 2).

The yearly analysis revealed that GT data had strongest correlations with ILI and virological surveillance in 2009. The correlation coefficient for the association between GT for *Fever* and ILI was 0.82,and 0.66 between GT for *Cough* and positive test rates of influenza virus in this pandemic year. The correlation coefficients describing the association between GT for *Fever* and ILI surveillance were statistically significant in all four years. Even though the correlation between the GT for *H1N1* and ILI surveillance could be observed in the overall data, there was no single year when the correlation was statistically significant (Table 1).

**Table 1:** Annual Pearson's Correlation Coefficients between Google Trends and CDC surveillance data from 2008 to 2011.

Year	Datasets	Google Trends					
		Influenza	Common cold	Fever	Cough	H1N1	Influenza A
In 2008¶	ILI	NA	−0.16	0.81*	−0.09	NA	NA
In 2009	ILI	−0.12	0.22	0.82*	0.14	0.09	−0.02
	Virologic	−0.01	0.74*	0.23	0.66*	−0.26	0.63*
In 2010	ILI	−0.47	−0.22	0.71*	−0.50	−0.25	−0.23
	Virologic	0.68	−0.13	0.63*	−0.28	0.37	−0.50
In 2011	ILI	NA	−0.08	0.64*	0.24	NA	NA
	Virologic	NA	0.62*	−0.10	0.43*	NA	NA

¶ There is no weekly virologic surveillance data in 2008.

* *P*<0.05.

### Lag Correlation analyses

A lag time of 0 weeks gave the highest correlations between influenza surveillance data and GT for most search terms. A decrease in correlation coefficients with lag time could be observed for GT search terms of *fever*, *Common cold* and *cough*. The peak of these search trends occurred at the same time as that of time series data from CDC surveillance. However, the trends for the search term ‘*Influenza A*’ starting from the preceding 1 week had a slightly higher correlation (*ρ* = 0.66, *p*<0.05) with virological surveillance data in 2009 compared with a lag time of 0 weeks (*ρ* = 0.64, *p*<0.05) (Table 2).

**Table 2:** Pearson's Correlation Coefficients in 1–2 lag weeks between Google Trends and CDC surveillance data from 2008 to 2011.

Surveillance Data	Google Trends					
	Influenza	Common cold	Fever	Cough	H1N1	Influenza A
ILI, 2-week preceding	0.22	−0.05	0.59*	−0.01	0.61*	−0.10
ILI, 1-week preceding	0.20	0.03	0.68*	0.01	0.58*	0.03
ILI, 0-week lagging	0.14	0.06	0.73*	0.02	0.51*	0.15
ILI, 1-week lagging	0.07	0.07	0.72*	0.03	0.41*	0.18
ILI, 2-week lagging	−0.01	0.02	0.66*	−0.01	0.32*	0.15
Virologic, 2-week preceding	0.19	0.45*	0.31*	0.17*	0.25	0.49*
Virologic, 1-week preceding	0.25	0.46*	0.35*	0.19*	0.28	0.57*
Virologic, 0-week lagging	0.22	0.46*	0.35*	0.20*	0.25	0.64*
Virologic, 1-week lagging	0.13	0.41*	0.33*	0.20*	0.23	0.66*
Virologic, 2-week lagging	−0.04	0.31*	0.26*	0.17	0.15	0.56*

* *P*<0.05.

## Discussion

Web search logs have been effectively applied to help monitor influenza activities in many developed countries [2], [9], [14], [15]. Carneiro and Mylonakis suggest that using Google Trends for disease surveillance is better suited in developed countries [3], which have large populations of internet search users. However, despite China's status as a developing country, it has nearly 400 million internet users [16]. In Guangdong province over 40 million people have access to the Internet, accounting for 40% of the total population. Such a large population of web users should provide reliable data for search-term influenza surveillance in the province.

The statistical analysis undertaken indicates temporal correlations between some Google Trends in Chinese language and influenza epidemics. Both ILI surveillance and influenza virus surveillance in Guangdong are correlated with Google Trends data statistically, but the statistical significance for the search terms are different. At a provincial level, the GT search term data for *fever* and *cough* are significantly correlated with conventional surveillance data. Interestingly, GT for *fever* is more highly correlated with ILI surveillance while *Cough* and *Common cold* are more associated with virological surveillance. Fever and cough are the two most common manifestations in influenza cases. Those patients with ILI are usually aware of these two presenting complaints at the pre-diagnosis stage of influenza. Thus these two search terms should be sensitive to influenza epidemics.

The local habits of web searchers can be influenced by users' level of education and their cultural and language backgrounds [3]. Pelat et al [17] reported that the Google search for *Influenza* in French is highly correlated with ILI surveillance data (*ρ* = 0.82, *p*<0.001). Valdivia and Monge-Corella [18] also found that queries for *Influenza* in Spanish show a significant correlation (*ρ* = 0.70) with national ILI surveillance data. In contrast, the GT term for *Flu* in Chinese was not statistically correlated with the official surveillance data, suggesting that this professional term in Chinese hardly reflects the influenza activity. This study also found that Google Trends have a lower correlation with weekly positive rates for influenza virus test than they do with ILI percentages which is consistent with Ortiz et al [5] who argued that Google Flu Trends does not do a good job in estimating laboratory-confirmed influenza cases. As a type of syndromic surveillance, the ILI surveillance system is designed to collect data from likely influenza cases in order to signal actual influenza activities. Neither fever nor cough is a specific symptom caused only by influenza virus infection. Our results indicate that search terms describing symptoms of influenza rather than those professionally used key words can better reflect actual influenza epidemics in the south of China.

Guangdong reported 9896 laboratory-confirmed cases of H1N1 and 36 deaths in 2009 [19]. The high prevalence of H1N1 influenza increased online health-seeking activity. However, health care seeking behavior and internet search behavior might be different and change over time during a pandemic period. GT in 2009 were more strongly associated with surveillance data than those in the other years. It appears that those affected patients typed in some topical words for this pandemic period, like *Fever* and *Influenza A*, to search health information on the web. The increasing public concern and media interest also raised the level of internet searching. Taking *H1N1* for example, as a search term, it became a hot word for internet search in 2009. However the yearly correlation coefficient shows the GF term *H1N1* had no association with influenza during the pandemic period, which is in contrast to the overall coefficient. In Guangdong, GT for *H1N1* spiked in May 2009, but the actual local H1N1 incidence peaked in November. The *H1N1* search trends did not reflect the actual H1N1 pandemic activity. A possible reason for the 5 months gap between the two peaks is that the mass media started to report H1N1 events when this novel influenza virus was first imported into Guangdong in May 2009. At that time, continuing news reports, outbreak briefs and health publications on the web heavily influenced *H1N1* search trends. Hence the May peak was associated with fear and information gathering in the community and the November peak of actual cases was the result of the actual progression of the spread of the disease within the province.

It is believed that increasingly patients are using web searches for health information prior to seeing a doctor [6]. Hence internet search trends can reflect actual epidemics earlier than conventional surveillance. One advantage of Google Trends is that data can be obtained earlier, more easily and at little cost, while the CDC published surveillance reports usually need one to two weeks for laboratory tests and data analyses.

However, publicly reporting official surveillance data can also raise awareness of health risks and increase internet searches. Possibly, because of this interaction, our study did not find any significant improvements in correlations between Google Trends and influenza surveillance with time lags, in contrast to previously reported findings [3], [6], [17], [20].

### Limitations

There are several limitations of this study. In terms of correlation coefficients, our results are lower than those in prior studies that compared Google Flu Trends data to traditional surveillance data [5], [7]–[9]. First, our study obtained only 4 years of Google Trends data, and for some search terms, there was insufficient data collected over the study period. The correlation between influenza virus surveillance data and GT data was limited to only two years of data beginning in 2009. A sore throat is also a common symptom in ILI cases, but Google fails to calculate its trends in Chinese due to insufficient proportions of this term in the total searches. Additionally, users may enter synonyms that we did not collect. More search terms might need to be investigated and correlated with standard surveillance data. Another limitation is that our analyses likely over- and underestimated some correlations. It is difficult to identify to what extent search trends are generated by true cases. Cook et al [21] suggested that search data may work well for diseases with less media exposure as media reports will probably drive more non-patients to increase their web search, which can influence search trends but not reflect the actual disease activity. The correlations found in this study between CDC surveillance data and GT for *H1N1* and *Flu* in 2009 are consistent with this finding.

### Conclusion

In conclusion, this study has shown Google Trends data using Chinese search terms are generally well correlated with conventional methods of surveillance. Google Trends, especially those related to ILI symptoms could be used as a complementary source of data for influenza surveillance in south China. However care should be taken when there is high media reporting of a particular influenza illness, which can bias internet search trends. The development of search-term based surveillance is still in its early phase. While considering the impacts of publicity, research in the future should develop new tools using search trends in Chinese language to estimate local disease activity as well as assist in detecting early signals of outbreaks.
